# Environment Degradation, Health Threats, and Legality at the Artisanal Small-Scale Gold Mining Sites in Indonesia

**DOI:** 10.3390/ijerph20186774

**Published:** 2023-09-17

**Authors:** Ami A. Meutia, Dianto Bachriadi, Nurfitri Abdul Gafur

**Affiliations:** 1 Research Institute for Humanity and Nature, 457-4 Motoyama, Kamigamo, Kita-ku, Kyoto 603-8047, Japan; dianto@chikyu.ac.jp; 2 Research Center for Environmental and Clean Technology, National Research and Innovation Agency, Building 820 Geostech, South Tangerang 15341, Banten, Indonesia; nurfitri.abdul.gafur@brin.go.id

**Keywords:** ASGM, gold mining, Indonesia, environmental degradation, health threats, legality

## Abstract

Artisanal Small-scale Gold Mining (ASGM) activities, despite offering numerous economic incentives, inflict negative impacts on the environment and public health due to the use of mercury or cyanide. This study aims to compare three ASGM locations in Indonesia in terms of environmental impacts and potential health problems in the community. As part of this research, field surveys at three ASGM illegal locations with different conditions, observations, literature studies, and interviews with the community and stakeholders were conducted. At each of the survey sites, the potential threat to public health due to the use of mercury was determined to be high. Additionally, many of the environmental impacts detected were deemed to have reached a high-alert stage, in some cases even posing a level of extreme danger. Based on these results, it has become clear that a form of mining management which reduces the negative impacts on the surrounding environment and community health is needed. The suggestions put forward in this paper, including the call for greater control of ASGM, may also be applied in other developing countries which suffer from similar problems and conditions.

## 1. Introduction

Environmental and potential health problems caused by ASGM are occurring throughout the world, most notably in developing countries [1,2,3,4,5,6,7,8]. This is particularly the case in Southeast Asia [1], and most especially in Indonesia, which is regarded as a resource-rich country. On one hand, the mining sector is one of the main sectors that sustains the national economy, by generating foreign exchange for the country, contributing to the Gross Domestic Product (GDP), and providing the largest proportion of energy resources, while absorbing labor employment from communities around mining sites [9]. On the other hand, this extractive economy, especially gold mining, is the cause of many problems. These range from environmental degradation to legal issues, labor exploitation, and various human rights violations, as well as social and health problems [10,11,12,13,14,15,16]. The legality/illegality of small-scale mining activities, sometimes called informal mining operations, has become a pressing issue. Of the approximate 900,000 Indonesian miners working at ASGM companies [17], most would be categorized as illegal miners, operating amidst the estimated 2645 total illegal mining locations across the country [18]. Today, ASGM in Indonesia is deemed illegal for two reasons: first, because it is being performed without a formal permit to operate, and second, because many of them use illegal substances such as mercury for amalgamation processes.

There is an abundance of recent studies on the negative impacts of ASGM activities on the environment and health in gold-rich countries including Indonesia. Some of the topics these studies focus on include: environmental degradation due to inadequate regulation, the discrepancy between economic interests and maintaining environmental sustainability [8,10,19,20,21,22,23,24], bad waste management systems [25,26,27], mercury-use and its impact on human health [1,4,6,7,28,29,30,31], as well as strategies/initiatives for improving the capacity of ASGM to perform good mining practices [26,32]. Regarding health threats, a study on the global use of mercury in ASGM showed that around 25–33% of ASGM workers globally suffer from moderate “chronic metallic mercury vapor intoxication” and around 8.7–12.6% of Indonesia’s small-scale gold miners have the potential to experience a disability as a result of this intoxication [28].

There is undoubtedly a close relationship between legality and environmental problems caused by ASGM activities. For instance, in Kenya, a country where 97% of ASGM activities use mercury for amalgamation processes, 95.5% of ASGM activities operate illegally and 94.3% do not conduct environmental assessment for their mining activities [8]. Meanwhile, the high degree of heterogeneity in the ASGM workforces, from the Philippines and Indonesian cases, on one hand, shows that legal mining operations are not an easy thing to implement, especially with regard to protecting miners and other related workers [11,33,34]. On the other hand, even if a mining site is legally operated, this does not necessarily guarantee that miners will operate in a cleaner way, as observed in Columbian ASGM activities [35].

The illegality of the ASGM operations in Indonesia is detrimental to the government’s interests, including taxes and levies, and to some extent, their practices act in direct opposition to existing regulations [36,37]. As a result, however, the actions taken by the government tend to only include discriminative policies against ASGM and lack governmental encouragement to reform ASGM, thereby limiting the opportunity to improve the situation or allow for the implementation of “green” mining practices [11]. Moreover, the most significant problem is that ASGM practices in Indonesia are detrimental to local communities because they pollute the environment, due to the disposal of untreated mine waste [19,25], resulting in threats to local people’s health [15,27,29]. Perhaps what is most troubling is the resulting onset of social conflict between local and migrant miners, between the community and legal mining companies, and between illegal miners and the local government [10,38].

Despite the burgeoning rise in Indonesia-centered case studies on ASGM activity, environmental degradation, and health issues, such studies have generally been carried out as separate analyses, in terms of both the issues at hand and the locations of the studies. There are hardly any studies that present a holistic picture of these issues from various perspectives, comparing legal, environmental, health, and social issues, while also proposing solutions to these problems comprehensively. This paper aims to try and fill that gap.

## 2. Materials and Methods

**Figure 1:**
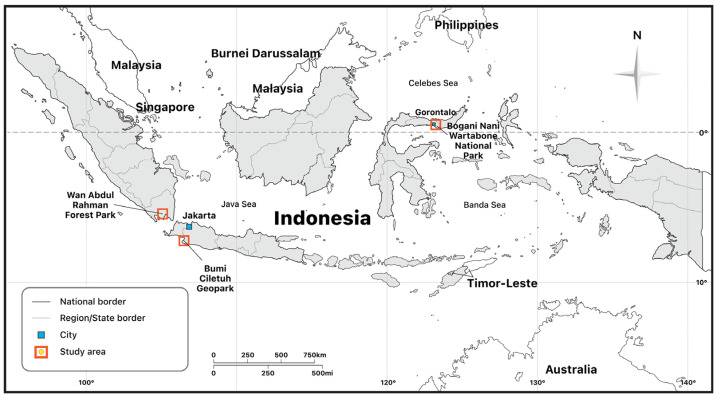
Field survey locations: Tulabolo Village near Bogani Nani Wartabone National Park, Bone Bolango Regency, Gorontalo Province; Harapan Jaya and Bunut Seberang Village near Wan Abdul Rahman Forest Park, Pesawaran Regency, Lampung Province and Cigaru, Kertajaya Village near Bumi Ciletuh Geopark, Simpenan sub-district, Sukabumi Regency, and West Java Province.

Our research was conducted with other teams in the same project. Our research team gathered data using a variety of techniques, including field surveys, observation, and questionnaires, followed by in-depth interviews, as well as transformational learning activities performed in Bone Bolango Regency. We distributed questionnaires to 200 households. Health examinations were also conducted for participants, which were then followed by more in-depth interviews. This paper’s data were mainly collected through literature and documentation studies (for legality aspects), field survey observation (for environmental aspects), and interviews with stakeholders and participants, using in-depth interview methods. The research locations are Tulabolo Village, subdistrict East Suwawa, Bone Bolango Regency, Gorontalo Province in Sulawesi Island, where the existence of illegal ASGM overlaps with the gold mining area of the contract of work area of PT. Gorontalo Minerals, Harapan Jaya and Bunut Seberang Villages, District Kedondong and Way Ratai, Pesawaran District, Lampung Province in Sumatera Island, which is known for the many small mining companies operating side by side with illegal ASGM, and in Cigaru of Kertajaya Village, Simpenan Sud-district, Sukabumi Regency, and West Java Province, where almost all of the miners are employed via an illegal ASGM cooperative which is supported by the Indonesia Small-Scale Mining Association (Figure 1). These locations were selected with the consideration that there are differing conditions inherent to each of the areas that play host to illegal mining.

## 3. Results

### 3.1. Legality/Illegality Aspects of Gold Mining in Indonesia

#### 3.1.1. Legal Mining

To carry out mining activity in Indonesia, business entities, cooperatives, and individuals must apply for a license called an IUP (Mining Business Permit) or IPR (Small-scale Mining Permit). If a mining operation does not have either an IUP or IPR, the mining activity will be categorized as illegal in nature. Originally, based on the Government Regulation No. 96/2021 on the Implementation of Mineral and Coal Mining Business, the two permits could only be issued by the Ministry of Energy and Mineral Resources. However, in order to shorten the mining business licensing procedure, since 2022, both permits have been delegated to the provincial government (based on the Presidential Regulation No. 55/2022). However, both permits should be carried out at locations previously determined by the ministry, namely, the Mining Areas (WP: *Wilayah Pertambangan*) for the IUPs and the People’s Mining Areas (WPR: *Wilayah Pertambangan Rakyat*) for the IPRs. Those permits, before they can be issued, also require an approval both from the local government and the Ministry of Forestry if the actors will use forested areas.

The problem of granting legality to ASGM in Indonesia is that the policymakers do not take into account the fact that ASGM has been practiced in the country long before the provision of IPRs. Frequently, the countermeasures that are applied against the ASGM take the form of police enforcement, sometimes even taking on a militaristic approach, which ultimately ends up never solving the problem or reducing ASGM activity. This approach denies ASGM any opportunities for improvement, both in maintaining the environment and providing benefits for regional economic development. Similar experiences were found in Ghana [39]. Even with the current stipulation in which IPR must be carried out in People’s Mining Areas (WPR: *Wilayah Pertambangan Rakyat*), this regulation is considered discriminatory for small-scale miners. This is because there are very few WPR areas compared to designated areas for corporate-based large-scale mining activities, and such areas often do not contain gold deposits (thereby not meeting the economic scale of the business) to work on [10].

#### 3.1.2. Illegality of ASGM and Environmental Problems

Illegal ASGM is defined as mining activities carried out by legal entities or individuals who do not have an IPR license for extracting and transporting minerals including gold [9,40]. The government has paid special attention to illegal ASGM practices, either due to the various negative impacts of their operations, including those related to social, economic, and environmental life, or because of their operations taking place within the Mining Areas (*Wilayah Pertambangan*) that have been designated for use by corporate-based large-scale mining operations [10,38]. But it must be noted here that even though ASGM absorbs more labor forces and, in fact, has the potential to contribute significantly to the local/community economy, the Indonesian government tends to prioritize large-scale corporate-based mining rather than mobilize its resources to improve the conditions of ASGM and its capacities [10,11]. According to the government’s recent data, in 2020, there were 143 gold mining permits issued, which covered around 1.6 million hectares of land across the country, compared to only 8 small-scale gold mining permits, which covered only around 17.8 hectares of land [41]. Of the many ASGM activities spread across the numerous provinces of Indonesia, only one legal ASGM can be found in Gorontalo Province, one of the provinces where our research was conducted, while not a single legal ASGM site exists in the other two provinces [41].

From an ecological point of view, illegal ASGM causes environmental damage, destroys forests, and threatens fauna and flora. It can also lead to environmental disasters and disruptions in the productivity of agricultural land and plantations and may result in river water turbidity and water pollution. Moreover, mining activities that transform the landscape are very likely to become involved in environmental issues, including issues related to carbon emissions, ex-mining holes (voids), the use of explosives in mining activities, and non-compliance with environmental quality standards. From a regulatory perspective, illegal mining violates Law no. 3 of 2021 concerning Amendments to Law (UU) no. 4 of 2009 concerning Mineral and Coal Mining.

In general, former illegal ASGM land that used the open-pit method leaves behind voids and puddles after the miners cease their operations, meaning that the land can no longer be utilized for other purposes. Almost all illegal ASGM operations do not have access to acid mine drainage treatment facilities. This has the potential to contaminate river water.

The implementation of illegal ASGM also generally ignores occupational safety and health. Many violations have occurred, such as the use of non-standard equipment, not using personal protective equipment (PPE), not having air ventilation in underground mines, and a lack of infrastructural support in underground mines.

**Figure 2:**
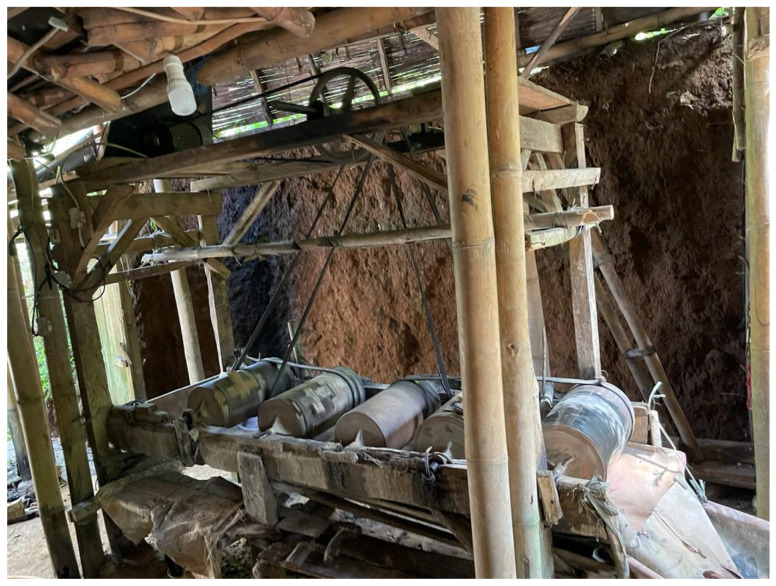
Grinding gold ore with mercury in cylinders. Location: West Java Province.

The most dangerous threat to health is the use of mercury by illegal miners. Mercury is one of the most essential substances in illegal ASGM, as it is necessary for the purpose of efficiently extracting as much gold as possible in a short span of time. The process of grinding gold ore with mercury in cylinders, which is commonly carried out by miners, only requires up to 24 h (Figure 2). Mercury can be used to extract gold under a variety of field conditions. Processing gold with mercury is considered more economical than other alternative techniques.

However, the practice of processing gold with mercury by small-scale gold miners is undertaken without public knowledge and concerns about the dangers of mercury. ASGM miners make direct contact with mercury without any protection, meaning that the risk of mercury exposure is exacerbated. The surrounding environment and communities that live around the location of illegal mines are also at risk of being exposed to the dangers of mercury.

In the illegal ASGM locations of Bone Bolango Regency, Gorontalo Province and Pesawaran Regency, Lampung Province, besides mercury, cyanide is also used to obtain gold in the secondary process after the use of mercury. Cyanide is also used in several mining areas where mercury is no longer permitted to be used in the process of collecting gold. In Indonesia, the use of cyanide in the mining process has been regulated via the Regulation of the Minister of Environment no. 23 of 2008. Cyanide is one of the chemicals that are used in extracting gold in both the large- and small-scale mining industries. As a result, gold mine workers and communities surrounding mining sites are very likely to experience cyanide poisoning.

Especially in illegal ASGM, most miners do not pay attention to the proper handling of cyanide waste. Usually, after all the processing activities are complete, the wastewater is simply dumped into the ground, often flowing directly into the river. Even in cases where mercury is no longer used, the implementation of cyanide management to minimize risks to human health and the environment is one of the main challenges being faced. Based on existing studies [29,30,31], most cases of poisoning in mining are caused by mercury (Hg), but there are also cases of cyanide poisoning, although they are still comparatively rare.

The social impacts of illegal ASGM activities include the hindering of regional development because they are not in accordance with the area planning (RTRW), the triggering of horizontal conflict within the community, the creation of vulnerable conditions and security disturbances in the community, as well as damage to public facilities. From the government’s perspective, illegal ASGM also has an impact on the country’s economy because it has the potential to reduce both tax and non-tax state revenues. In addition, it serves as a trigger for socio-economic disparities, causes fuel shortages, and has the potential to increase the prices of goods that people need.

Will making ASGM activities legal address the negative environmental and health impacts they cause? And are illegal miners willing to change? We will address these important questions in the Section 4 below.

### 3.2. Environmental Degradation of Several Gold Mining Locations

In this study, the authors conducted a survey at three locations (Figure 1) of illegal ASGM. Each location was chosen because of its distinct characteristics. In this research, the authors conducted interviews with miners, local communities, stakeholders, and people closely associated with illegal ASGM in the area while also measuring levels of mercury found in water sources used in daily life, such as well water, underground water, drinking water from the drinking water company, as well as hot water sources used by the surrounding community. The results in this study mostly take the form of descriptions from the results of interviews and field observations in these areas. Table 1 describes the conditions of the three locations.

#### 3.2.1. Suwawa Timur District, Bone Bolango Regency, Gorontalo Province

In Gorontalo Province of Sulawesi Island, there are many mining companies. Traditional gold mining in Gorontalo originated from the Dutch colonial area around 1899, which was located at Buladu District, North Gorontalo. Traditional gold mining by individuals in Bone Bolango Regency has been performed for a long time, since 1940, during the colonial reign [11,23].

**Figure 3:**
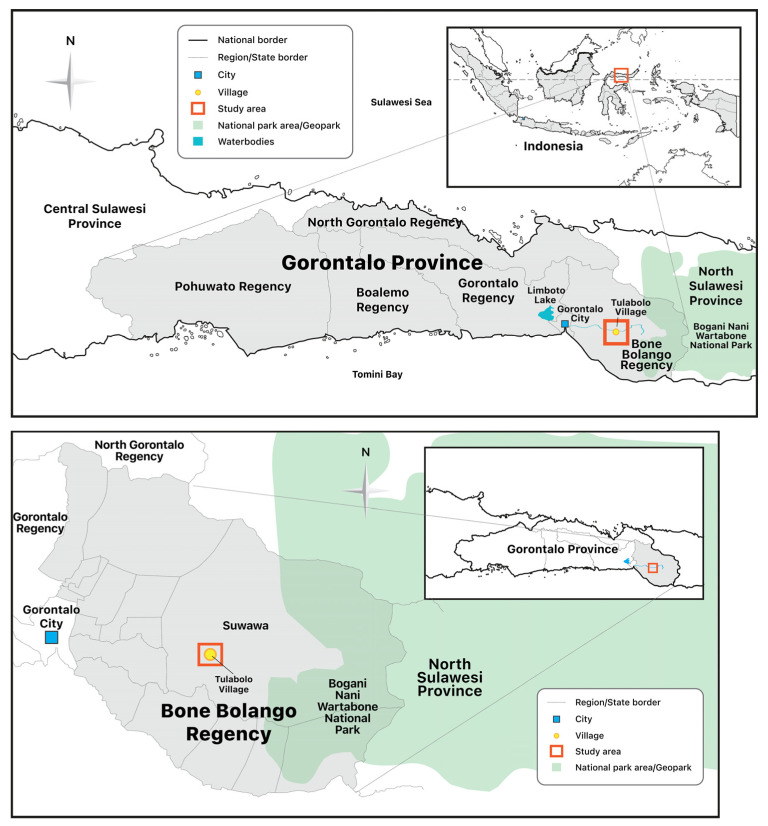
Tulabolo Village, Bone Bolango Regency, Gorontalo Province.

**Figure 4:**
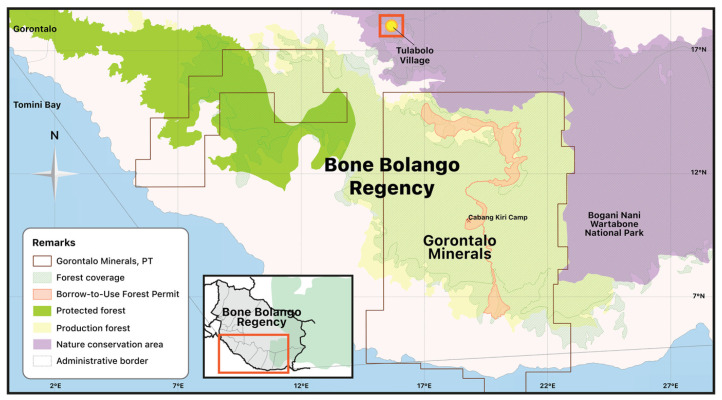
Mining Permit area of PT. Gorontalo Minerals. Source: © Auriga|Data source WebGIS ESDM, WebGIS KLHK, RBI BIG.

Bone Bolango Regency, where Tulabolo Village is located, has an area of 1984.31 km², and the number of inhabitants as of the year 2021 was 164,277 people (Figure 3). Bone Bolango Regency plays host to several areas that have become mining sites. One such area of gold mining operations is the Suwawa Timur sub-district, where the Tulabolo Village and other mining-belt villages are located. Many ASGM mines operate in this sub-district together with one large-scale mining company, namely, PT. Gorontalo Minerals. (Figure 4). The total concession area of PT. Gorontalo Minerals, after a reduction from the original designation due to the existence of a protected forest and a national park, is around 24,995 hectares. The original concession area of this large-scale mining operation, as determined by the government in 1998, is 51,579 hectares [10].

With regard to the actual conditions of ASGM sites in East Suwawa, from an environmental hygiene perspective, they tend to suffer from poor management systems for waste, be it the waste from extraction activities or other daily human waste. In terms of work safety, almost all illegal ASGM companies here do not meet the standards of work safety, and it is suspected that these workers have been exposed to mercury. This is based on the results of health examinations and in-depth interviews conducted, including several complaints from mining workers regarding health problems, such as frequent headaches, difficulty swallowing food or drink, blurred vision, decreased body weight (thinness), feeling thick in the legs and arms, and sometimes diarrhea. Such health problems are thought to be due to exposure to mercury.

Illegal ASGM activities also result in various environmental changes, including changes in the landscape, changes in the habitats of flora and fauna, changes in the soil structure, changes in surface and groundwater flow patterns, and so on (Table 2). Ignorance of the environmental impact of miners coming from outside the area has also exacerbated environmental damage in the gold mining area in Bone Bolango.

Table 3 summarizes the results obtained by the authors and other secondary sources during the years 2013 to 2021, investigating the mercury concentration in the Bone Bolango areas. The table includes comparisons of the mercury content in several samples taken by the research team, measured against certain threshold standards determined by the authoritative institutions. Any measurement of mercury concentrations that is considered below the threshold has been highlighted in a green font color. If the measurement results range from below the threshold to above the threshold, indicating potential contamination or a status of high alert, then this is denoted by a yellow font color. Any measurements that exceed the threshold are symbolized with a red font color. As can be seen in the following table, water sources used in daily life by the community in Bone Bolango, such as well water, ground water, etc., contained low concentrations of mercury, while the results of previous studies in river water showed high concentrations. Meanwhile, both soil and sediment show signs of having potentially entered a polluted state. From a medical perspective, potential impacts on physical wellbeing in Bone Bolango were discovered, with alarming concentrations of mercury being found in hair and blood. There were also indications of impacts upon plant life in the location. Edible plants like rice were found to contain alarming concentrations of mercury. Low concentrations of mercury were discovered in the drinking water of Bone Bolango, including wells and springs (10 locations). Likewise, the fish that are widely consumed by the public in Bone Bolango are still safe for consumption because the mercury concentration is comparatively low. Although the concentration is still below the threshold, carnivorous fish accumulate mercury in their bodies and often contain higher levels of mercury accumulation.

Bone River, one of the rivers in Gorontalo Province, is subject to the greatest threat from mercury pollution. As elucidated by other researchers about ten years ago, Bone River was found to have been polluted with mercury [29,46], with this pollution apparently having continued until the present day (Table 3). The main cause of this is the presence of traditional gold mining as a producer of mercury waste, which is discharged into the stream of Bone River. This mining activity existed even before Indonesia’s independence, so it is feared that the poisoning process that occurs chronically in the people who use the Bone River water source must be of serious concern. The problem is that Bone River serves the main domestic and industrial needs of Gorantalo and supports a large proportion of the aquatic life in the region.

The existence of PT. Gorontalo Minerals’s (GM) concession, which later resulted in contention with local ASGM companies, can be traced back to 1970, when PT Tropic Endeavour Indonesia held a mining contract of work in Suwawa, which expired in 1986. This company only performed geological surveys and exploration (pre-production) activities. In 1988, a new permit was issued for PT Antam, a state-owned mining company, in collaboration with BHP, a US-based mining company, to continue exploration activities in PT Tropic’s former concession. But since 1991, exploration has been halted due to the problems caused by its location overlapping with that of a protected forest [10]. A year later, in 1992, several individual local miners who previously joined the exploration survey began to dig at an abandoned exploration site [11]. A gold rush occurred in Suwawa following the success story of these ASGM pioneers. However, in 1998, a new mining concession was issued by the government for PT Gorontalo Minerals (GM), a new joint venture formed by BHP and PT Antam. GM ownership changed again following the takeover of BPH shares by the International Mineral Corp., a subsidiary corporation of the PT Bumi Resources Tbk., in 2005. Since then, conflict between GM and local miners including the communities of the Tulabolo Village and other mining-belt villages occurred (Table 1) due to the more active survey and exploration activities conducted by GM [10,11,47].

The conflict also took place during the peak period of COVID-19 in 2021 [47]. As the results of our observation in 2022–2023 show, the conflict is still ongoing. However, there are no detailed steps that have been taken, meaning that the problem has been dragging on up until the present day. Now, the land that has been exploited by the community since the 90s is included in the company’s mining concession area. In this way, people who have always mined there are now considered illegal because they are operating inside of the mining company’s concession.

Communities who have been mining for a long time, of course, want to be able to continue their activities. Some of them have asked their mining sites to be removed from the GM’s concession so that they may begin plans to operate legally. Some others are willing to cooperate with the company, seeking to form a joint mining operation [11]. Mediation between the company, ASGM officials, and the community has been carried out by the local government, with unsatisfactory results: GM persisted to hold its currently existing concession, with no more reduction, and has no interest to cooperate in any form with local miners. Meanwhile, the government’s offer to relocate the ASGM operation into the designated Small-scale Mining Area (WPR) located near the GM’s concession was rejected by the ASGM officials, as they claimed that there are no gold mineral deposits in that area [10].

The existence of the mining company also threatens the environment and the people of Bone Bolango, posing a danger to residential areas, water sources, agriculture, and fisheries. The community asked the provincial government to evaluate any mining companies that have started production operations [10]. The Gorontalo government must therefore take into consideration the farmers around the mining areas, as it is these farmers who are most affected.

The presence of the company’s gold mine is also considered to have damaged the surrounding environment and created a more severe disaster risk. Not long ago, there were flash floods in Bone Raya, resulting in landslides that claimed lives, and threats of even worse disasters. On 7 September 2020, in Bone Raya District, there was a flash flood and landslide caused by high rainfall, which claimed a great deal of lives and property. It can therefore be imagined that should the mining company fully operate by digging forests and land with open-pit mining, the threat of disaster could become even worse.

The open-pit mining method that will be used by the gold mining company in Bone Bolango Regency will not only cause deforestation and exacerbate the impacts of climate change, but it will also have an impact on the flora/fauna in the forest (Table 3) [10]. This is especially a threat to the typical species that can only be found on Sulawesi Island.

It is thought that the presence of a mining company that is very close to the Bogani Nani Wartabone National Park (TNBNW) could have a significant impact on the biodiversity in that area. This protected area, which is the largest terrestrial national park in Sulawesi, covering an area of 282,008.757 hectares, boasts high biodiversity and is an important habitat for 317 typical Sulawesi species [48]. The wild animals that have long lived in these locations will be the most affected, mainly due to the increase in noise pollution caused during the main operating stages.

#### 3.2.2. Pesawaran Regency, Lampung Province

**Figure 5:**
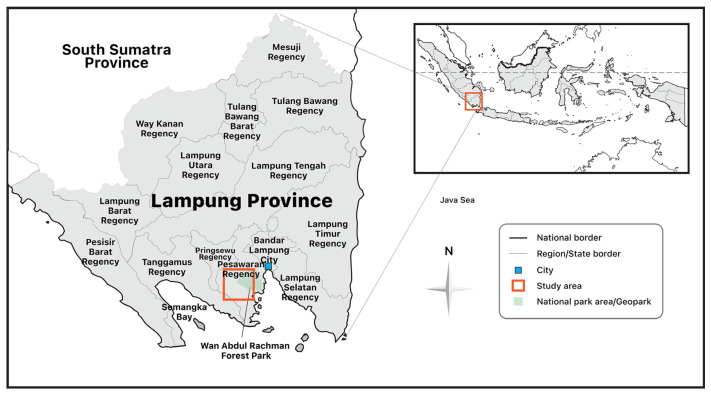

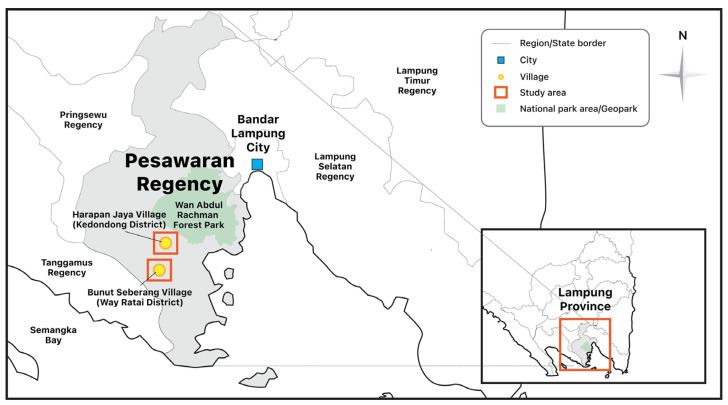
Illegal mining location in Harapan Jaya and Bunut Seberang Village, Pesawaran Regency, Lampung Province.

Lampung Province is an area that has abundant natural resources and energy sources, as well as minerals. Based on the directory of mining companies in Lampung province, there are many small mining companies that have concessions of around hundreds to thousands of hectares (Figure 5).

Pesawaran Regency is one of the regencies in Lampung Province that is host to a lot of gold mining and ranks second after South Lampung in terms of the number of mining and quarrying companies. In Pesawaran Regency itself, there are six companies operating. Administratively, the total area of Pesawaran Regency is 1173.77 km^2^. The population of Pesawaran Regency in 2020 was estimated to have reached 474,926 people.

One of the areas in Pesawaran is a natural resource center and gold mining center called Bunut Seberang Village, situated in Way Ratai District (Figure 5). Bunut Seberang Village is host to several ASGM areas and has multiple gold refining points located around community settlements. Gold mining activities in this village began in the 1990s, expanding to the present day. The abundant energy and mineral resources in Bunut Seberang Village were utilized by the local community to open a gold processing business that employed local workers.

The population of Bunut Seberang Village in 2020 reached 3079 people. Of this population, many residents made a living via gold mining work in processing plants, as motorcycle taxi drivers who take stones from the mountains, or as stone crushers.

The gold processing process carried out by residents in Bunut Seberang Village starts with taking stones from the mountain, which are then broken into small sand sizes before being placed in a rotating machine mixed with mercury. The mixture process is continued in a spindle machine for one day and one night until it becomes sludge. The sludge is then put into a vat to produce dark black carbon and burned until it becomes a silver sphere. Next, the silver sphere is washed with hard water to extract the gold. This process results in large quantities of mercury evaporating into the water bodies, soil, and plants adjacent to the community’s living space.

Table 4 shows that in Pesawaran, river water and soil contained high concentrations of mercury. Because of polluted environmental conditions, it is not surprising that the local government in Pesawaran closed several small mines. Potential impacts upon plant life were also found in Pesawaran. The mercury concentration among edible plants was considerably high. The mercury concentration was measured to be at a high alert level in trees, whose wood is utilized as building materials and for making furniture.

In the village of Bunut Seberang, atmospheric mercury pollution moves in a south and southeasterly direction, with this movement being influenced by the factors of wind speed and wind direction. It was noted that that in Pesawaran Regency, not only has water and soil been polluted by hazardous chemicals, but the air has also been polluted because of the ASGM activities. Traces of pollutants in the air stick to the bark of the trees, allowing them to be sampled and measured [49,50,51], as shown in Table 4.

**Table 4:** The impact of mercury pollution in Pesawaran Regency.

Location	Year (Sampling)	Sample Type	Mercury Concentration	Guidelines	Ref.
Pesawaran Regency	2020	Soil	0.26–28.9 mg/L	0.005 mg/L Indonesian Government Regulation No. 82 year 2001	[52] (data from the same project team)
		River water	0.08 – 14.1 mg/L	0.005 mg/L Indonesian Government Regulation No. 82 year 2001	
Pesawaran Regency	2022	Fruit tree bark *Tamarindus indica*, *Persea americana*, *Annona muricata*	0.82 mg/g, 0.41 mg/g, 0.58 mg/g	0.03 ppm Hg threshold	[49] (data from the same project team)
Pesawaran Regency	2022	Tree bark *Magnolia champaca*, *Swietenia mahagoni*, *Pterospermum acerifolium*	1.01 µg/g, 0.38 µg/g, 0.37µg/g	Exceed 10 ppm: Hyperaccumulators of Hg, 1 ppm: Toxic and does not exceed 0.5 mg/kg Director General of National Agency of Drug and Food Control No: 03725/B/SK/VII/89	[50] (data from the same project team)

Green: low (mercury concentrations of all samples are below the threshold). Yellow: alert (there are mercury concentrations that are below the threshold and some that are above it). Red: high (mercury concentrations of all samples are above the threshold). Blue: guidelines have been added because they were not found in the original research source material.

In Pesawaran Regency, many small gold mining companies employ local people who originally mined illegally. Because these companies provide jobs to the community and build village infrastructure and Islamic boarding schools, also providing many other assistances to the local community, there is no conflict between the company and the community (Table 1). Even though there was no conflict over the mining location between the company and the community, residents urged the government to close several gold mining companies that polluted their environment because of ASGM activities around their settlements. Such incidents occurred several times in several villages in Pesawaran Regency (Table 5), where there was mass poisoning of up to hundreds of affected residents. The suspected cause of the poisoning was due to the pollution of the river, which is a source of clean water for the village community. The pollution is allegedly related to the existence of gold mining activities around the river. The company’s activities are suspected to have violated hazardous and toxic materials (B3: *Bahan Berbahaya dan Beracun*) waste management procedures because there are no drains or catchment ponds for running water, nor is there a temporary storage place for B3 waste. In recognition of its danger, mercury is gradually being replaced by cyanide in the amalgamation process. However, as noted in Pesawaran Regency, this cyanide waste being directly dumped into the environment without any processing is also causing harm to humans and other living things. One case of cyanide poisoning, caused by the substance being dumped into the river in 2019, has received sufficient attention from the community and the government, leading to a temporary suspension of operations for small mining companies.

The gold processing business in Bunut Village, Way Ratai District not only employs men but also employs women to crush the rocks that have been brought from the mountain. Females often take up work as stone breakers in Bunut Seberang Village because of economic pressure and the need to increase their income. The average income contribution of female workers in the gold mining area in Bunut Seberang Village ranges between 26 and 50% of the total household income [53].

Adjacent to Way Ratai is the Tahura (People’s Forest Park) Wan Abdul Rachman (WAR) landscape on Register 19, Pesawaran District, Lampung, with an area of 22,249.31 hectares. Although illegal ASGM is not found in the Tahura WAR area now, the illegal ASGM activities adjacent to the Tahura WAR can indirectly threaten the existence of fauna and flora living there, including around 82 species of birds, 26 species of mammals, and 266 species of trees [54]. The need for environmental care in Tahura WAR is especially pressing, because according to the spatial plan of Lampung Province, a prospective mining location has been identified in Tahura WAR.

#### 3.2.3. Cigaru, Kertajaya Village, Simpenan Sub-District, Sukabumi Regency, West Java Province

**Figure 6:**
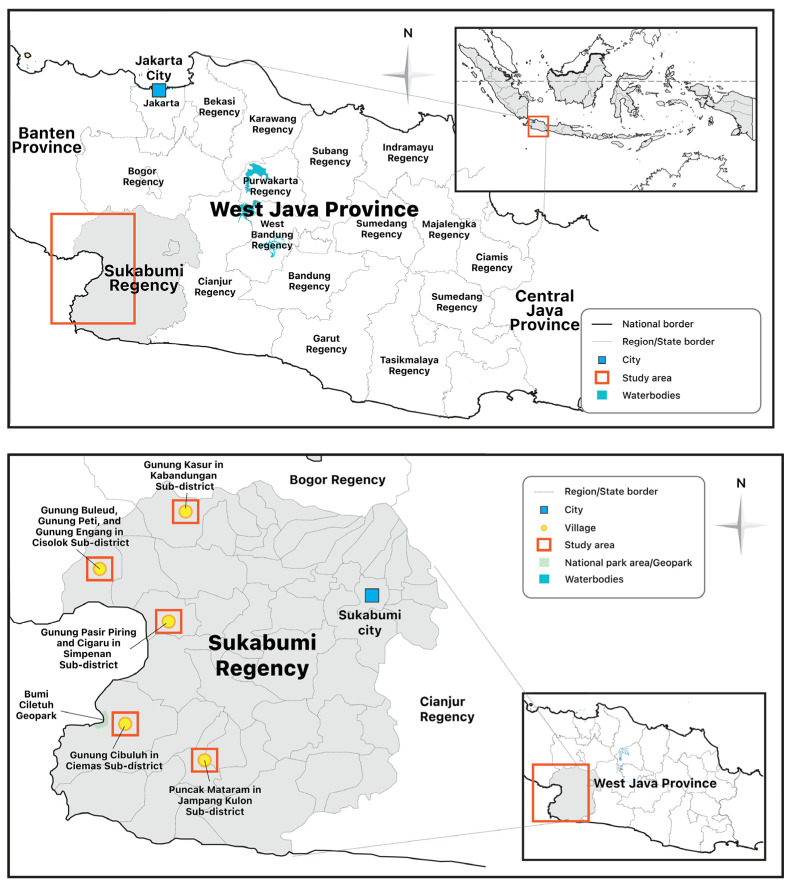
Cigaru Kertajaya Village, Simpenan sub-district, Sukabumi Regency, West Java Province.

ASGM in Sukabumi District can be traced back to the 1920s, when the Dutch colonial authorities encouraged gold mining activities in this area. Now, hundreds of ASGM groups are spread across seven areas in four sub-districts. The locations are Gunung Kasur in Kabandungan sub-district, Gunung Buleud, Gunung Peti, and Gunung Engang located in Cisolok sub-district, Gunung Cibuluh in Ciemas sub-district, Puncak Mataram in Jampang Kulon sub-district, and Gunung Pasir Piring in Cigaru Simpenan sub-district (Figure 6). Various techniques are used by ASGM to extract gold ore in these areas, such as panning, digging mine-holes, and small-scale open-pits. The use of mercury and cyanide is the main method by which the gold recovery process takes place here.

Both mining operations conducted by the ASGM and the large-scale company PT Golden Pricindo Indah (GPI) in Cigaru, Sukabumi Regency have contributed to the degradation of the local environment. The difference is that ASGM contributes significantly to soil, water, and air pollution due to the use of mercury and cyanide. Meanwhile, the company, which does not use mercury at all, and has a waste processing installation, contributes to the environmental problems via its tailings that contain cyanide. The company’s open-pit method, which uncovered a “green layer” and stripped the top layer of the earth’s crust, has led to wider environmental problems. While the hole-digging method used by the community has “only” damaged a relatively small area of the environment in/around the mine-holes, the environmental damage that has occurred around the Cigaru mining area has resulted in serious river and coastal sedimentation. Mud and tailings carried by several small rivers, whose sources are located in the mountains around Simpenan sub-district, have made these small rivers and the large rivers at the mouth, such as the Cibuntu, Cidadap, Cikaso, and Cileteuh Rivers, very sludgy. Other serious consequences of pollution in these rivers and coastal areas are decreasing, even the loss of aquatic biotas.

In Sukabumi (Table 6), the mercury concentration of river water was found to be below the threshold in 2018 [55,56], but from the results of a recent study, the concentration of mercury was found to have increased to very high levels [57]. Whereas the mercury concentration in river sediment is high, river water showed a low concentration. The low amount of mercury in the water samples may be a consequence of speciation (the occurrence of mercury in low-solubility phases): because river water flows, the concentrations of mercury in the water tend to be low. However, the concentration of mercury in sediments in the same river can be high because mercury settles at the bottom of the river. In soil and plants, concentrations of mercury were counted at alarmingly high levels. The high concentration of mercury found in hair samples in Sukabumi indicated that the spread of mercury in this region has reached the point that it is posing a potential negative impact on health.

Our observation focuses on mining operations in the Cigaru area at Simpenan sub-district (Figure 6), where both ASGM companies and a large-scale mining company, namely, PT Golden Princindo Indah (GPI), operated in proximity (300-hectare concession). Both have been operating on unproductive plantation land, with the land rights (Commercial Use Rights or HGU: *Hak Guna Usaha*) being held by PTPN VIII, a state-owned plantation company. The difference is that GPI, which started mining exploration in 1995, obtained an IUP in 2011. Meanwhile, the ASGM companies, which have been operating there since the 1990s, did not obtain a permit, even though they had applied for one several times. According to one of the ASGM leaders, excavation in the new mining blocks is necessary because, in the current area, many holes have reached a depth of more than 80 m. As a result, besides being dangerous, the potential for gold is reduced as well.

In 2009, when the government issued the IUP for around 300 hectares to GPI, protests from the ASGM groups occurred [58]. The area designated by the IUP also covered several parts of the local ASGM operation areas, including some areas that have been targeted by the community for use. GPI offered to cooperate with the ASGM actors, who had dug in those areas beforehand. As part of this offer, ASGM miners would be allowed to continue their operations, but the gold produced would be sold to the company. This cooperation offer was rejected. Smallholder miners preferred their mining areas to be removed from the company’s IUP area, resulting in the breakdown of agreement discussions.

In 2015–2016, a group of ASGM companies organized via a cooperative, namely, Sukabumi Miners Group (KPS: *Kelompok Penambang Sukabumi*), as members of the Indonesian Small-scale Mining Association (APRI), applied to the government for small-scale mining permits (IPR). This attempt received support from the local government and several ministries, including the Ministry of Energy and Mineral Resources (ESDM). Although the ESDM Ministry was ready to provide the IPR as requested, the state-owned plantation company refused to release their land for use by small-scale mining operations. Due to this, the KMS-KPS proposal failed. At that time, KPS had already built a small complex for small-scale gold mining operations, which was recognized as being able to fulfill some requirements necessary to implement a good mining practice, including the ability to maintain the environment in good condition, build waste installation processing units, and establish a system to manage the occupational health and safety of laborers.

Without an IPR, KPS ASGM was then expelled from their mining area in the PTPN VIII plantation location. Some of them then began to dig mine-holes around settlements, on private lands, and built in-house mercury-based gold processing facilities in the middle of villages. The disposal of the mercury-laden tailings around the settlement is a common occurrence at this site. In addition, ASGM in Kertajaya Village also continued its business in other blocks in Cigaru, such as in the Tengki Block located in the unproductive JA Wattie plantation.

Not so far from Simpenan sub-district, there is a geological heritage site named Cileteuh Geopark, which was designated as part of the UNESCO Global Geopark Network on 12 April 2018. The area covers 126,100 hectares, spanning eight districts and 74 villages. There are at least 62 geological sites included, consisting of exotic scenery, rocky islands, waterfalls, sea caves, beaches, rare rocks, fossil sites, and hot springs. Gold mining activities in Simpenan sub-district, both those operated by the ASGM and the large-scale mining company, have threatened the existence of this area both as a geological heritage site and as a primary tourism destination. The area around the Ciletuh River, especially the Ciemas District, contains rocks, which are evidence of the collision of the Eurasian continental plate with the Indian–Australian Ocean during the Cretaceous Age, about 50 million years ago. The rocks also act as evidence pointing to the Ciletuh area being the oldest plain in West Java.

Mining in the Ciletuh Geopark area can have a negative impact on the environment because the area is a water catchment area. The environmental damage that occurs includes the onset of drought, landslides, floods, and even threats to the health of the population. Recently, an illegal gold mine in the Perhutani land area of Cibuluh Block, Ciemas Village/District, Sukabumi Regency was closed by the Sukabumi authorities due to numerous problems occurring at the location, namely, the fact that it often claimed lives and damaged the environment. There are hundreds of former illegal gold mines that have been closed for similar reasons.

## 4. Discussion

### 4.1. Lesson Learned from Three Locations of Illegal ASGM

**Figure 7:**
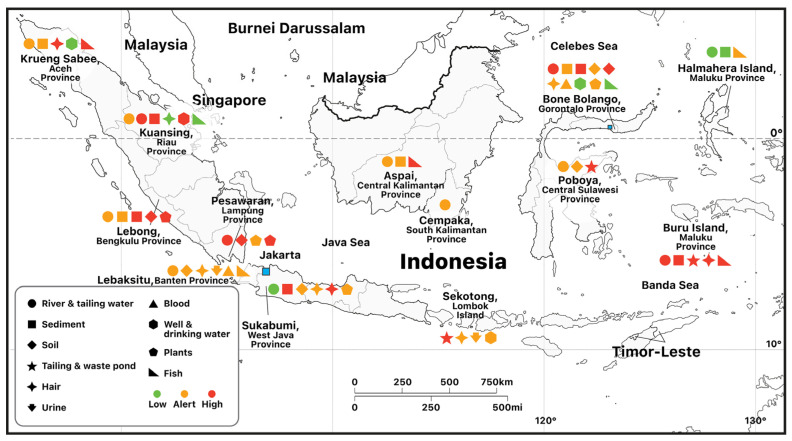
Mercury concentration levels sampled around ASGM in Indonesia in 2010–2021, compiled based on primary research and secondary sources.

What are the real effects of illegal mining on the environment and public health? To answer this question, we added quantitative data for our sample sites, drawn from the results of secondary-source studies undertaken in the past ten years at several gold-mining sites in Indonesia [59,60,61,62,63,64,65,66,67,68,69,70,71,72,73,74,75,76,77,78,79,80,81,82,83,84]. The environmental impacts and health threats from illegal mining operations can be seen in Figure 7. As can be seen, there were few places where the mercury concentrations in the water, sediment, and soil were below the guideline-designated threshold (colored green). Samples from the river water [27,52,69,77,78,81] indicated that the mercury content had exceeded the threshold. The same condition was found in the Philippines, Thailand, and Colombia [1,5]. This is also the case for the results of samples taken from humans, including hair, blood, and urine. With regard to this potential impact on health, we can see from the map that the mercury concentration in hair, blood, or urine is indicated as being in the “alert” status. There are even studies showing that the mercury content in hair exceeded the designated threshold [56,72]. Miners in the Philippines and in Colombia also showed mercury concentrations in blood, urine, and hair above WHO thresholds [1,7]. A mercury concentration in human hair is a direct consequence of ASGM activities and is an indication of a potential risk to human health. The mercury concentration in edible plants such as rice and fruit also reached the “alert” stage. The concentrations found in fish vary, as the mercury content in fish depends on the type of fish being sampled. As people should avoid eating types of fish that are known to contain high levels of mercury. In some areas, the mercury level in drinking water was measured to have reached the “alert” status and even “high”. This indicates the potential for health impacts or that health impacts are already occurring, further necessitating the imperative to improve environmental conditions. In interviews with several respondents, we noted multiple responses complaining of tremor symptoms, headaches, etc., all of which are potential symptoms of mercury intoxication. However, further research is needed to ensure that the symptoms are the direct result of mercury intoxication and not of other causes.

Illegal ASGM activities carried out conventionally, directly, or indirectly have had a huge impact on the level of public health. It was found that many people were affected by health problems caused by the pollution of waste chemicals flowing into the river. Direct impacts include the emergence of various kinds of skin diseases experienced by miners and the people who live around the mining site, as can be seen at Pesawaran Regency (Table 5). In Harapan Jaya village, there was even a mass case of almost 200 people being negatively affected by the consumption of fish from polluted rivers. This happened on multiple occasions (Table 5). Sources of water contaminated with mercury waste also become unfit for consumption. In addition, the death of fish in the mining environment occurs, as well as the emergence of diseases experienced by residents due to the consumption of polluted fish from the river near the mining sites. This case occurred at Tulabolo and Harapan Jaya Village.

Research in Buyat Bay has shown that mercury and cyanide contents have been found not only in rivers but also in the sea [85]. At Kao Bay, North Halmahera in Maluku Province, mercury, and cyanide levels were found in fish bodies, which exceeded the WHO standard threshold [74]. As listed in Table 3, there were also incidents of livestock being killed by cyanide poisoning in Pesawaran. Other studies reported that mercury contamination was also present in livestock [86].

The research results showed that the impact of illegal ASGM carried out by individual miners is harmful to the environment around mining sites. In this way, mining activities carried out without a license can be said to have a negative impact on the surrounding environmental ecosystem.

Damage to environmental ecosystems caused by the mining activities of mining companies that have licenses is included as part of the Environment Impact Analysis (AMDAL), so they have an obligation to carry out mining land reclamation programs under intensive supervision. Conversely, illegal ASGM activities such as in Tulabolo, Bunut Seberang, and Kertajaya Village, which operate unlicensed, do not have an obligation program to reclaim their ex-mining land and cannot be monitored intensively.

However, environmental problems and health threats are less considered as risks in mining operations, especially but not limited to the case of ASGM [87]. As a result, pressure is needed from external sources through policy changes and, frequently, public pressure to render it as an obligatory matter which requires attention. It is not surprising that miners, mining operators and related stakeholders prioritize the continuity of their business, rather than paying attention to environmental risks. In general, focus is drawn towards technical, operational, economic, and financial risks, which take precedence over risks that are political, legal, social, environmental, health-related, or managerial in nature [88].

Besides uncontrolled environmental pollution, there have been various natural disasters occurring as a result of mining activities, such as in Bone Bolango and Sukabumi Regency, where landslides often led to the death of miners. Therefore, the necessity to change the mindset of the community regarding gold mining activities is imperative. This also includes the urgent need to implement good mining practices that are environmentally sound. While fostering environmental awareness within the traditional mining sector, local governments must actively supervise mining activities, strictly and consistently applying various regulations in the field of mining and environmental management, and must help traditional mining activities comply with legal principles, especially with regard to the protection and management of the living environment [89].

There is an urgent need for outreach to the local community regarding the harmful effects of using mercury in the gold mining process, which will be fatal to the health of the local community and the surrounding environment. However, for financiers, miners’ safety is not the primary concern. This is because, as found from the results of interviews with funders, the provision of protective clothing can lead to financial losses, as, sometimes, miners will use such clothing to hide gold pilfered from the pits.

In Pesawaran Regency, the small gold mining company was temporarily closed in 2019 and remains closed today (2023). It can be seen here that small mining companies that use cyanide are having difficulties obtaining permission to operate again, in the case that their mine wastewater is not undergoing treatment. An appropriate level of technology is required to treat cyanide wastewater produced by ASGM. Supposedly, if the company is allowed to operate again, then it must comply with the standard operation procedures (SOP), where there is monitoring and evaluation of mining work. This process, included as part of the refining of precious metals and the disposal of waste, should arguably be subject to strict regulation, ensuring that there are no harmful risks to the environmental ecosystem and society.

In the Bunut Seberang Village, we would propose that the local government should provide outreach to mining areas, so people who work in mining areas are aware of the risks of working in mining, the safety of working in mining, and the impact of mining on the surrounding environment. This is because women who work in mining areas are less aware of the risks and lack of safety involved in mining.

### 4.2. Complexity of Legality/Illegality and Governmental Actions for Transforming ASGM

Efforts to deal with illegal mining have been carried out over a long period of time, with no appropriate solution in sight. This is partly due to the complexity of the factors that have led to the rise of illegal ASGM activities, including: a lack of government support and facilities, discriminative treatment; a lack of economic opportunities that provide better incomes for the miners, coupled with limited employment opportunities in non-mining sectors; financiers’ control of the operation to gain more profit and backing from the local elites making them reluctant to move toward legal mining operations [10,11]. In every form of mining activity, including ASGM, in general, there are three major barriers to implement cleaner technologies and cleaner production practices, namely, legislative, technological, and economic barriers [89]. An expanded government role is needed to break these barriers.

Perspectives regarding the legalization/formalization of ASGM itself vary. There is a stark difference in perspectives between the government and small-scale miners, where the government mostly perceives formalization as being related to control, taxes, and state revenue [10,23]. On the other hand, ASGM actors, in general, do not view legalization as a necessity, due to the complicated bureaucratic procedures and costs involved. Even if they gain formal status, this does not make it any easier for them to access credit from formal financial institutions such as banks [90]. On the contrary, they prefer to work in illegal or informal ways, since it is easier for them to have support from powerful local elites and have capital provided to them from such parties [11].

From the perspective of ASGM, many stakeholders are, however, willing to follow tax provisions, as well as other policies and regulations related to regional economic development and environmental sustainability, if their existence is recognized and formalized [10,11]; see [91] for a similar perspective from Peruvian ASGM. In this context, to achieve more effective conditions allowing for the reduction in negative impacts caused by ASGM activities, any possible policies and efforts for ASGM formalization should consider the nature of the ASGM activities themselves, which cannot be equated with large-scale mining activities [33]; see also [34] for a comparison from the Philippines ASGM perspective.

Regarding the procedures, it has been proven that the transfer of legalization authority from regencies to the Ministry of ESDM, then back again to the region at the provincial level, had little effect on the number of illegal mining sites becoming legal [17].

The Indonesian government has quite often attempted to control illegal ASGM using police repression and military-like actions at the sites, with such incidents occurring several times in East Suwawa of Bone Bolango and in Sukabumi Regency, where the government closed the mine hole and arrested illegal gold miners [10,11,92,93]. Preventive efforts to halt illegal ASGM, as well as repressive measures in the form of the expulsion and forced evacuation of miners from illegal ASGM sites, have been carried out many times but have always been met with challenges from local communities and the miners. As a result, the number of illegal ASGM sites has not decreased but is, on the contrary, increasing. One of the triggering factors is the shortage of job vacancies, which has led to mass unemployment [11,12,94]. Even though some individuals may be aware that gold mining is difficult work which damages the environment and public health, a lack of alternative options forces them to continue.

Perhaps the most inhibiting factor originates from the applicable laws and regulations causing changes in licensing authorities in Indonesia, as well as existing regional regulations that have not been put into effect properly. This has taken the form of law enforcement officials who have not effectively implemented regulations under supervision. There is also the non-compliance factor of the person in charge of the mining business in complying with the contents of the license granted by the government, as well as applicable laws and regulations such as not involving the surrounding community in the AMDAL preparation process. Additionally, there is the factor of the lack of public awareness of the environment and the lack of knowledge about enforcing environmental law, as well as the factors caused by the limited facilities and inadequate means of enforcing environmental law in Indonesia [95]. The above factors might be the key to the question of why local and central governments seem unable to control illegal ASGM, let alone unwilling to encourage cleaner production methods [89] or green and climate-smart mining [32].

As for the question of why there are so many illegal ASGM activities in Indonesia, two reasons why ASGM actors do not pursue mining licenses are that the procedure to obtain a license involves complicated bureaucratic procedures that take a long time to complete and limited formally designated areas for small-scale mining activities [10]. Besides that, they must have an environmental permit from the Ministry of Environment and Forestry as a means by which to prevent environmental damage caused by mining activities and post-mining reclamation efforts. Also, under strict governmental regulation, small-scale mining permits (IPR) are only given to residents. In fact, many miners come from other areas because members of the local population lack mining experience, or the outsiders are only participating as financiers. Gold miners who come from other regions are usually invited by financiers. Workers from outside the area are preferred by the funders because they do not return home often, allowing them to concentrate more on their labor. In contrast, residential miners often return to their village because it is close to the mining location. As such, the residential status can be seen as one of the difficulties in fulfilling the requirements from the government.

In order to deal with illegal ASGM, the central government took steps in relation to illegal ASGM activities in licensed areas (concession areas), including the transfer of illegal mining activities to non-community-based mining activities through community involvement/development and empowerment programs by license holders; partnership efforts or the granting of Mining Services Business Licenses (IUJP); as well as coordination with license holders to be able to shrink a portion of the permit area where illegal ASGM activities are carried out so that they can work in Small-scale Mining Areas (WPR) where the ASGM can process the IPR.

The central Indonesian government received many opinions from local governments and from business actors, who stated that local governmental management would be more effective. As a result, Presidential Regulation 55 of 2022 has been in effect since last April, and the submission of OSS (online single submission) will facilitate the licensing process in the regions. In addition, this delegation will slightly ease the tasks of the Directorate General of Mineral and Coal. Previously, many local authorities stated that they were powerless to act, since mining permits are a matter for the central government. With this delegation, even if only partially, local governments can better supervise their regions and control illegal mining. Moreover, there are several methods of settlement that can be carried out by the government, companies, and miners’ associations to help illegal gold miners escape illegal conditions.

Another community-based activity to help local miners out of illegal mining activities is the search for alternative forms of income. Various suggestions were offered to the community via the creation of participatory activities which could lead to alternative livelihood sources [96] and improve community health [97]. Participatory community health practices can potentially help the community to maintain their health and the health of miners routinely. Because the community is suspected to have been exposed to harmful chemicals, the Bone Bolango Regency Government has undertaken plans to conduct health checks on residents in and around the mining location.

### 4.3. Suggestions

Are there any good examples of illegal mining? To answer this question, we conducted research in three locations, and the results did not find a good example of illegal mining management that has a positive impact on the environment, society, and government. The results of the research show that each illegal mining location has specific problems that must be solved according to the conditions of each respective region.

We propose that mining stakeholders formulate a concept of “green and fair ASGM”, a parallel concept to “green and climate-smart mining” [32], which we refer to here as the Responsible Mining Community (RMC). In an exploration of the challenges in implementing “green and climate-smart mining”, Jiskani et al. [32] found that governance and regulatory, technical, and operational, and cognitive and social aspects among stakeholders, as well as financial and economic conditions and functional/organizational aspects, are challenges that must be overcome. These challenges align with the Responsible Mining Community (RMC).

Initially, RMC was an idea developed by the Indonesia Small-scale Mining Association (APRI) in order to receive legal recognition from the government for small-scale mining operations in Indonesia. We believe that the RMC should go beyond just a formalization: it should be an ideal ASGM operation, which essentially builds a foundation for good ASGM governance. RMC implementation should carry several principles, such as: institutional building, environmental sustainability, safety, and healthy working conditions, while strengthening the local economy, green products and markets, as well as social justice [11].

**Figure 8:**
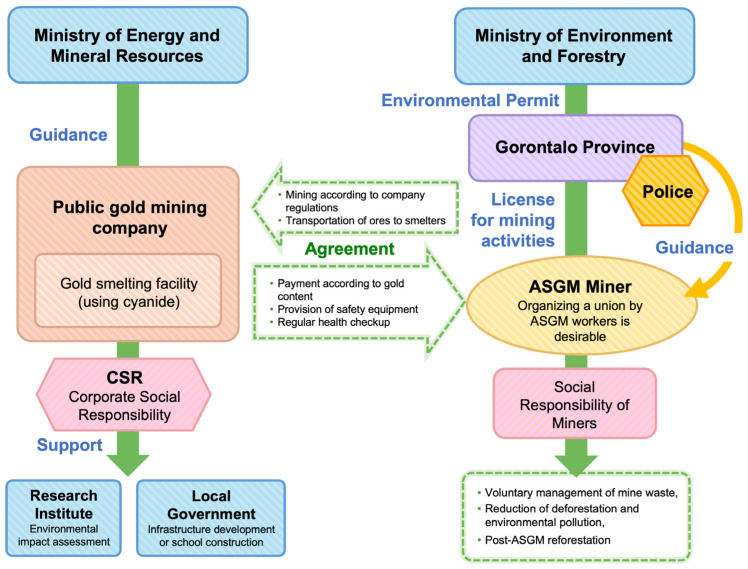
Suggestion for good mining practice at Tulabolo Village. The blue color indicates the government agency related to licensing process or supports it. The purple color is for province which is decider for licensing. The pink color indicates social organization. The apricot color indicates the company. The dark orange is a legal authority and the beige color is the miners’ organization.

In this sense, the formation of the RMC still requires a more comprehensive study of various ASGM practices in Indonesia; see [98] for an argument from China’s experience. At the same time, a government’s commitment is needed to resolve the issue of legality/illegality in a comprehensive manner while considering the nature of the ASGM practice itself. Meanwhile, with regard to conflict issues between ASGM and large-scale mining companies, such as those that occurred in East Suwawa of Gorantalo and Simpenan of Sukabumi, we encourage strategic steps towards joint operations. Figure 8 shows a suggestion for good mining practice in the case of Tulabolo Village, in which there is a conflict between the mining company and community. This chart explains our suggestion for illegal miners to join a forum/union and ask the Ministry of Environment and Forestry for environmental permits. In doing so, mining companies can then cooperate with illegal ASGM organizations and provide areas that can be mined by them. The company would therefore have a conditional agreement with illegal ASGM organizations, allowing illegal ASGM to be controlled to a greater extent and for the company to accommodate its mining products from illegal ASGM.

In the case of illegal ASGM located at a counter-work location owned by PT. Gorontalo Minerals, the community has been there for decades, meaning that the government must find a way to compromise. For instance, this could take the form of encouraging local governments to make it easy for the community to obtain licenses in the mining sector. The government would be expected to provide opportunities for illegal miners to take part in business partnership programs with mining companies holding Mining Business Permits (IUP) so that former illegal gold miners whose activities are within mining company concessions can become subordinates. So, for mining business activities with certain concessions, this would be mutually beneficial for both parties, the communities and mining companies.

So far, the local government can be deemed partly responsible for the damage caused by illegal miners in reclaiming ex-mining areas. It is better if the costs of the reclamation of the mining environment and public health checks are borne by mining actors, which can be large and small companies or organizations/groups of miners and even individuals. Its application can be in the form of environmental and health preservation through taxes or corporate social responsibility (CSR) from large mining companies.

Figure 8 is a proposed chart for the mining case in Tulabolo, Bone Bolango. Further study is needed to prove whether this suggestion can be successfully applied there and/or elsewhere.

## 5. Conclusions

This research shows that impacts and threats caused by illegal mining regarding the environment and health are very clear in the three locations. At all three locations, it has been found that the environmental impact of mercury concentrations is a cause for concern, even at low levels. Likewise, the impact on human health and food safety cannot be ignored. It is also clear from the field survey that the cause of the environmental impact is illegal ASGM. Solving the problems of environmental degradation and threats to human health posed by the increasing ASGM activities in Indonesia requires a comprehensive approach.

In addition to increasing the technical, managerial, and institutional capacity of ASGM in managing social and environmental risks and the resulting negative impacts, which would be possible via the implementation of Responsible Mining Communities (RMC), changes to the attitude by which government policies treat ASGM are needed. Reactionary or repressive measures, which have been the norm so far, are clearly insufficient. Although the concept of RMC can be viewed as a potential solution, in order to control illegal mining activities and to reduce their impact on the environment and public health, further in-depth studies which explore how to implement this concept are needed. Meanwhile, strategic moves to resolve conflicts that arise between ASGM and large-scale mining operations in the form of joint operations are a solution that is also worth considering. Although we have proposed a way to solve the problem of illegal ASGM in Bone Bolango Regency, further studies are needed to examine whether the proposal will be successful. If it is effectively carried out, it will not only solve the problem of illegal ASGM in Indonesia but also in other developing countries which suffer from the same issues.

## Figures and Tables

**Table 1:** Impacts of illegal ASGM on the environment in three locations.

Location	Description	Conflict	Environmental Effect
Tulabolo Village, Suwawa Timur, Gorontalo Province	Illegal ASGM inside and near National Park Bogani Nani Watobone. Side by side with a large mining company.	Between illegal miners and a large company	Flora and fauna at Bogani Nani Wartabone (intact). Fish in rivers.
Pasawanan, Lampung Province	Illegal ASGM side by side with many legal small mining companies.	No conflict; illegal miners support small companies	Forest Park Wan Abdul Rachman. Fish in rivers.
Sukabumi, West Java Province	Illegal ASGM joined the association of illegal ASGM miners. Operated within the large-scale plantation and Geopark.	Between the ASGM miners and plantation permit-holders	Bumi Ciletuh Geopark.

**Table 2:** Environmental conditions that are affected by Illegal ASGM at Tulabolo Village.

Affected Environment	Type	Condition
Forest	Protected forest	deforestation
	Limited production forest	deforestation
	Permanent production forest	deforestation
National Park	Fauna	threatened
	Flora	threatened
	Typical species of Sulawesi	threatened
River	Mak River	turbid
	Bone River	polluted
	Fish	killed
	Water	polluted
Land	Land	degraded
	Water table	decreased

**Table 3:** Mercury concentration in Bone Bolango Regency during the years 2013–2021.

Location	Year (Sampling)	Sample Type	Mercury Concentration	Guidelines	Ref.
Bone Bolango Regency	2021	Drinking water, well, and hot spring	0.02–0.03 µg/L	6 µg/L WHO	This research
Bone Bolango Regency	2018	Mining and tailing water	176–489 µg/L	6 µg/L WHO and 0.005 mg/L National standard for mining water	[27] (data from the same project team)
		River water (Bone River)	16–2080 µg/L	6 µg/L WHO and 0.002 mg/L (2 µg/L) Indonesian Gov.t Regulation No. 82 year 2001	
		Sediment at mining area Sediment at Bone River	ND–790 µg/g ND–57.9 µg/g	75 mg/kg PP 101 of 2014 and 0.174 ppm NOAA	
Bone Bolango Regency	2018	Soil	0–36 mg/kg	0.2 ppb US EPA	[42] (data from the same project team)
		Plant (*Pteris vittate*)	0–5.2 mg/kg	Exceeds 10 ppm: hyperaccumulators of Hg, 1 ppm: toxic and does not exceed 0.5 mg/kg Director General of National Agency of Drug and Food Control No: 03725/B/SK/VII/89	
Bone Bolango Regency	2018	Soil	32–131 mg/kg	0.005 mg/L Indonesian Gov. Regulation No. 82 year 2001 and 0.3 mg/kg PP 101 of 2014	[43] (data from the same project team)
		Dust (soil)	15–91 mg/kg	0.005 mg/L Indonesian Gov. Regulation No. 82 year 2001 and 0.3 mg/kg PP 101 of 2014	
Bone Bolango Regency	2022 (2018)	Bone River Sediment	9.5–86.3 µg/g	2 mg/kg	[44] (data from the same project team)
Bone Bolango Regency	2018	Nose (19 samples) and scalp hair (10 samples)	0.52 ppm–24.4 ppm	5 ppm high, <1 ppm safe and in between is the warning level	[45] (data from the same project team)
Bone Bolango Regency	2013	Fish (*Decapterus muroadsi*, *Laligo pealii*)	0.0298 mg/kg (average) 0.01880–0.04103 mg/kg	0.3 mg/kg National standard and Max 0.5 ppb (0.5 µg/g) WHO	[29]
		Blood (100 persons) 52 persons above national standard	2.92–378.90 µg/L	8 µg/L and <10 µg/L WHO	
		Hair (100 persons) 57 persons above national standard	0.48–260.2 µg/g	2.0 µg/g National Standard and toxicology threshold limit <1 µg/g normal,1–<5 µg/g alert and >5 µg/g high	
		Drinking water	0.000285–0.00065mg/L	0.001 mg/L Indonesian Gov. Regulation No. 82 year 2001	

Green: low (mercury concentrations of all samples are below the threshold). Yellow: alert (there are mercury concentrations below the threshold, and some are above it). Red: high (mercury concentrations of all samples are above the threshold). Blue: threshold standards that were not present in the original research source have been added.

**Table 5:** The impact of environmental pollution in Pesawaran Regency.

Month and Year	Village Name	Affected Environment	Suspected Chemical	Symptom	Affected
August 2010	Sinar Harapan (now, Harapan Jaya)	Cikantor River	Cyanide, etc.	vomiting, headache, painful/hot throat, shortness of breath, face very hot, dizziness, and heartburn	184 people 2 fainted
	Sinar Harapan (now, Harapan Jaya)	Fishponds	Cyanide	fish killed	18 ponds
	Sinar Harapan (now, Harapan Jaya)	Livestock	Cyanide	livestock killed	some places
March 2019	Babakan Loa	Cikantor River	Mercury	acute hives	some residents
February 2021	Bunut Seberang	River	Cyanide, etc.	itchy rash	some residents
September 2021	Harapan Jaya	Way Ratai River		feels itchy and fish killed	some residents, huge amount
	Way Kepayang	Way Ratai River		feels itchy and fish killed	some residents, huge amount
February 2023	Gunung Sugih	River		fish killed	huge amount

**Table 6:** The impact of mercury pollution in Sukabumi Regency.

Location	Year (Sampling)	Sample Type	Mercury Concentration	Guidelines	Ref.
Sukabumi Regency, West Java (Java Island)	2019	Hair (71 persons; 38 men, 33 women)	Men: 0.71–18 ppm Women: 1.5–24 ppm	Toxicology threshold limit: <1 µg/g normal, 1–<5 µg/g alert, >5 µg/g high	[56]
Sukabumi Regency, West Java (Java Island)	2018	Water	<0.00005 mg/L	0.002 mg/L PP No. 82 of 2001	[55]
		Sediment	101–196 mg/kg	75 mg/kg PP 101 of 2014	
		Soil	0.036–1.8 mg/kg	0.3 mg/kg PP 101 of 2014	
		Rice (plant)	<0.03–0.14 mg/kg	0.03 mg/kg SNI 7387:2009	

Green: low (mercury concentrations of all samples are below the threshold). Yellow: alert (there are mercury concentrations that are below the threshold and some that are above it). Red: high (mercury concentrations of all samples are above the threshold).

## Data Availability

Not applicable.
